# Outcomes of pediatric pilonidal disease treatment: excision with off-midline flap reconstruction versus endoscopic pilonidal sinus treatment

**DOI:** 10.1007/s00383-023-05629-1

**Published:** 2024-01-31

**Authors:** William G. Lee, Celia Short, Allen Zhong, Vanya Vojvodic, Andrew Sundin, Ryan G. Spurrier, Kasper S. Wang, Juan Carlos Pelayo

**Affiliations:** 1 Department of Pediatric Surgery, Children’s Hospital Los Angeles, 4650 Sunset Blvd, Los Angeles, CA 90027 USA; 2 Department of Pediatric Surgery, Cedars-Sinai Medical Center, 8700 Beverly Blvd, Los Angeles, CA 90048 USA; 3 Division of General and Thoracic Surgery, The Hospital for Sick Children, 555 University Avenue, 1526 Hill Wing, Toronto, ON M5G 1X8 Canada

**Keywords:** Pilonidal disease, Modified Limberg flap, Bascom cleft lift flap, Endoscopic, Minimally invasive, Recurrence

## Abstract

**Purpose:**

Pilonidal disease (PD) is marked by chronic inflammation and frequent recurrence which can decrease quality of life. However, debate remains regarding the optimal treatment for PD in the pediatric population. This study compares two recommended treatment approaches—excision with off-midline flap reconstruction (OMF: Bascom cleft lift flap, modified Limberg flap) and minimally invasive endoscopic pilonidal sinus treatment (EPSiT).

**Methods:**

Single-center retrospective evaluation of patients 1–21 years of age with PD who underwent either excision with OMF reconstruction or EPSiT between 10/1/2011 and 10/31/2021. Outcomes included were disease recurrence, reoperation, and wound complication rates. Comparisons were performed using Chi-square and Mann–Whitney U tests.

**Results:**

18 patients underwent excision/OMF reconstruction and 45 patients underwent EPSiT. The excision/OMF reconstruction cohort was predominantly male (44.4% vs 17.8% *p* = 0.028), with history of prior pilonidal infection (33.3% vs 6.7%; *p* = 0.006), and longer median operative time (60 min vs 17 min; *p* < 0.001). The excision/OMF reconstruction cohort had a higher rate of wound complications (22.2% vs 0%; *p* = 0.001), but lower rates of disease recurrence (5.6% vs 33.3%; *p* = 0.022) and reoperation (5.6% vs 31.1%; *p* = 0.031).

**Conclusion:**

In pediatric patients with PD, excision with OMF reconstruction may decrease recurrence and reoperation rates with increased operative times and wound complication rates, compared to EPSiT.

## Introduction

Pilonidal disease (PD), or pilonidal sinus disease, is an inflammatory disorder involving trapped hair or debris in the natal cleft [1]. PD is marked by chronic inflammation, sinus tract formation, secondary infection, and frequent recurrence [2]. In the United States, this affects 26 per 100,000 people with an increased frequency of occurrence in males and adolescents (14–25 years of age) [3, 4]. For adolescent patients with PD, the associated sequelae such as recurrent discharge or bleeding, pain, infection, abscess formation, and frequent healthcare encounters for treatment have been shown to significantly decrease quality of life and lead to increased absences from school or work [5, 6]. In rare cases, PD-related chronic inflammation can also increase the risk for squamous cell carcinoma formation in patients with compromised immune systems [7]. Thus, PD disproportionately affects the pediatric population and can have a significant impact on both physical and psychosocial wellbeing.

The primary goals of PD treatment are to excise all areas of disease, including undermining sinus tracts, while also minimizing post-operative pain, time away from baseline functional status, and the risk of wound complications (e.g., infection, dehiscence). Thus, the optimal treatment decreases the risk of recurrence and future reintervention, while also minimizing treatment-related morbidity. While prior studies have sought to determine the optimal treatment option, debate still remains regarding the most effective approach to achieving these goals [8–10]. Current treatment options include non-operative (e.g., hygiene/lifestyle modification, laser epilation), minimally invasive (e.g., sinusectomy/Gips procedure, endoscopic debridement), and excisional/reconstructive techniques (e.g., secondary healing, negative pressure wound therapy, midline primary closure, off-midline flap reconstruction) [11–15]. In recent years, minimally invasive treatment and excision with off-midline flap (OMF) reconstruction approaches have emerged as the recommended options due to favorable rates of achieving the aforementioned treatment goals [11, 16].

In the pediatric population, minimally invasive techniques are associated with lower rates of wound complications and a faster return to baseline functional status, but are limited by higher rates of disease recurrence and reoperation [11]. In contrast, excisional approaches with OMF reconstruction have demonstrated higher rates of wound complications and prolonged recovery time, with contradictory literature regarding long-term recurrence rates [11, 17]. In the adult population, excision combined with OMF reconstruction has been associated with decreased long-term recurrence rates [18]. This is attributed to the wide excision of diseased tissue and lateralization with flattening of the natal cleft achieved by OMF closure of the soft tissue defect [19–21].

In this context, prior studies have sought to compare excision with midline primary closure to minimally invasive techniques, or various off-midline flap reconstruction techniques to one another [8, 22–26]. However, in the pediatric population, little is known regarding direct comparison of minimally invasive endoscopic pilonidal sinus treatment (EPSiT) with excision and OMF reconstruction [11]. The objective of this study is to compare these two treatment approaches among pediatric patients with PD. We hypothesize that while excision and OMF reconstruction may lead to prolonged operative times and higher rates of wound complications, it may decrease PD recurrence and the need for reoperation—which suggests a more durable treatment option for the pediatric population.

## Methods

### Study design, patient population, and data elements

After Institutional Review Board approval was obtained (CHLA-21-00349), a single-center retrospective cohort study was performed. The study cohort included patients between 1 and 21 years of age who underwent surgery for PD between 10/1/2011 and 10/31/2021. Patients were identified using Current Procedural Terminology codes for pilonidal cyst excision (10,080–10081, 11,770–11772). Patients undergoing reoperation for recurrent disease or receiving management for an acute PD infection (e.g., incision and drainage, unroofing, antibiotics) were excluded. Only patients who underwent EPSiT or excision with OMF reconstruction (Bascom cleft lift flap, modified Limberg flap/MLF) were included in the analytic cohort. While prior studies in the adult population have suggested differing outcomes between these two OMF reconstruction approaches, validation in the pediatric population remains under investigation [27]. Thus, these two procedures were grouped together and compared directly to the MISTE approach. These procedures were performed as previously described [12, 20, 28–30] by multiple surgeons and procedure type was selected per surgeon preference. The EPSiT approach was performed by excising the draining sinus with a circular punch biopsy, followed by curette debridement and irrigation with a hydrogen peroxide-normal saline solution. A pediatric cystoscope was then used to inspect the sinus cavity and perform additional debridement with irrigation. In addition to operative management, non-operative management (e.g., hygiene modification, laser epilation) was also utilized for all patients per standard of care.

Data elements included patient characteristics (demographic, clinical) and clinical outcomes metrics. Patient demographic information included age, sex, and ethnicity. Clinical data included body mass index (kg/m^2^), comorbidities, history of pilonidal infection, operation type, total operative time, hospital length of stay, total narcotic use (in oral morphine equivalents), and the presence of post-operative complications. Follow-up data and post-operative outcomes were abstracted from the electronic health record and date of last follow-up was determined as the most recent evaluation by a surgical provider.

### Outcomes

The primary outcome was disease recurrence which was defined as the presence of active PD > 28 days after the initial operation. Secondary outcomes included the need for reoperation or the presence of wound complications (dehiscence, infection).

### Statistical analysis

Descriptive statistics were reported using frequency and proportions for categorical variables and median and ranges for continuous variables. Comparisons of categorical variables were performed using the Chi-square test, and the Mann–Whitney U test for continuous variables. Multivariate linear regression was performed to identify factors associated with disease recurrence. All analyses were conducted with two-sided significance and a significance cut-off level of 0.05. Analysis was performed using SPSS software v29.0.1.0 (IBM Corp., Armonk, New York, USA).

## Results

Over the 10-year study period, a total of 63 patients met the inclusion criteria with 18 patients undergoing wide excision with OMF reconstruction (Bascom cleft lift flap *n* = 10, MLF *n* = 8) and 45 patients undergoing the EPSiT procedure. The overall cohort had a mean age of 15.5 years (SD = 1.6) with the youngest patient at 12 years and eldest patient at 18 years. The majority of patients were of Hispanic ethnicity (*n* = 44, 69.8%) with a male predominance in the excision/OMF reconstruction cohort (44.4% vs 17.8%, *p* = 0.028) (Table 1).

**Table 1:** Patient demographic and clinical characteristics

	All patients (*n* = 63)	Excision with off-midline flap construction (*n* = 18)	Endoscopic pilonidal sinus treatment (*n* = 45)	*p*-value
Mean age, years (SD)	15.5 (1.6)	15.9 (1.2)	15.3 (1.7)	0.125
Male, *n* (%)	16 (25.4)	8 (44.4)	8 (17.8)	0.028
Ethnicity, *n* (%)				0.795
Hispanic	44 (69.8)	13 (72.2)	31 (68.9)	
Non-Hispanic	19 (30.2)	5 (27.8)	14 (31.1)	
Other/Multi/Unknown	0 (0)	0 (0)	0 (0)	
Median body mass index, kg/m^2^ (range)	29.7 (16.4–48.9)	31.3 (22.5–46.2)	28.8 (16.4–48.9)	0.474
Comorbidities, *n* (%)				0.386
Autoimmune/Inflammatory	6 (9.5)	3 (16.7)	3 (6.7)	
Cardiac	0 (0)	0 (0)	0 (0)	
Oncologic	1 (1.6)	1 (5.6)	0 (0)	
Metabolic/Endocrine	3 (4.8)	1 (5.6)	2 (4.4)	
Neurologic	3 (4.8)	0 (0)	3 (6.7)	
Psychiatric/Behavioral	0 (0)	0 (0)	0 (0)	
Respiratory	4 (6.3)	1 (5.6)	3 (6.7)	
None	46 (73.0)	12 (66.7)	34 (75.6)	
Prior pilonidal infection, *n* (%)	9 (14.3)	6 (33.3)	3 (6.7)	0.006

At median follow-up duration of 62 days, patients who underwent excision with OMF reconstruction experienced lower rates of recurrence (5.6% vs 33.3%, *p* = 0.022) and reoperation (5.6% vs 31.3%, *p* = 0.031) (Table 2). However, this cohort also had longer median operative times (60 min, range 35–97 min vs 17 min, range 6–68 min; *p* < 0.001) and wound dehiscence in four patients requiring wound care (Table 2). There was no difference in total post-operative narcotic use, time to recurrence, time to reoperation, or duration of follow-up (Table 2). Overall post-operative length of stay for all patients was ≤ 48 h with the majority of patients discharged within 24 h (*n* = 59, 93.7%) (Table 2). Four patients required admission for post-operative pain control, but were all discharged on post-operative day one.

**Table 2:** Clinical outcomes

	All patients (*n* = 63)	Excision with off-midline flap reconstruction (*n* = 18)	Endoscopic pilonidal sinus treatment (*n* = 45)	*p*-value
Median total operative time, minutes (range)	19 (6–97)	60 (35–97)	17 (6–68)	< 0.001
Post-operative length of stay, *n* (%)				0.87
< 24 h	59 (93.7)	17 (94.4)	42 (93.3)	
24–48 h	4 (6.3)	1 (5.6)	3 (6.7)	
> 48 h	0 (0)	0 (0)	0 (0)	
Median total post-operative narcotic use in oral morphine equivalents, mg (range)	8 (0.75–24)	9 (1.25–24)	7 (0.75–14)	0.763
Wound complication, *n* (%)	4 (22.2)	4 (22.2)	0 (0)	0.001
Recurrence, *n* (%)	16 (25.4)	1 (5.6)	15 (33.3)	0.022
Median time to recurrence, days (range)	124 (29–1357)	1357 (n/a)	82 (29–453)	0.2
Reoperation, *n* (%)	15 (23.8)	1 (5.6)	14 (31.1)	0.031
Median time to reoperation, days (range)	203 (50–1358)	1358 (n/a)	96 (45–567)	0.286
Median follow-up, days (range)	62 (7–1392)	64 (15–757)	56 (7–1392)	0.881

Multivariate linear regression identified younger age and longer follow-up duration as factors associated with an increase in PD recurrence (Table 3). Univariate regression analysis also evaluated the relationship between PD recurrence and additional variables—sex, body mass index, comorbidity type, history of pilonidal infection, total operative time, and presence of wound complications—and none were found to be associated with PD recurrence.

**Table 3:** Multivariate linear regression analysis of factors influencing disease recurrence in pediatric patients with pilonidal disease after operative intervention (*R*2 = 0.201)

Variable	Beta coefficient	Standard error (of beta coefficients)	95% CI for beta coefficient		*p*-value
			Lower bound	Upper bound	
Age	− 0.116	0.039	− 0.193	− 0.038	0.004
Follow-up duration	0.001	0	0	0.001	0.015

## Discussion

Treatment of PD in the pediatric population remains a challenge due to high rates of treatment-related morbidity and frequent recurrence resulting in repeated absences from school/work, decreased quality of life, and added psychosocial morbidity [5]. The breadth of treatment options alludes to the current lack of an optimal treatment modality to eradicate disease and mitigate morbidity. The observations from this comparative study highlight the benefits and limitations of two commonly utilized treatment options. While wide excision with OMF reconstruction demonstrated lower rates of recurrence and need for reoperation, the EPSiT approach required a shorter operative duration and exhibited a lower rate of wound complications. These findings reinforce similar outcomes from prior studies in the adult population and suggest that wide excision with OMF reconstruction is associated with increased early post-operative morbidity, but may provide a more durable treatment option for PD [24, 26].

In comparing the two operative modalities, excision with OMF reconstruction was associated with lower rates of disease recurrence and reoperation at a limited follow-up duration. Our observed recurrence rate after excision with OMF reconstruction was 5.6% which is consistent with previously reported rates ranging from 2.7 to 6.3% after excision with Limberg flap or MLF reconstruction [19, 20, 31]. We found this to be significantly lower than recurrence rates reported after minimally invasive treatment approaches, which have been previously reported at 13.2% at 5-year follow-up in the adult population and up to 38% at 28-month follow-up in the pediatric population [32, 33]. This may be accounted for by the larger area of excision and more complete eradication of the diseased tissue with the excisional approach. Thus, excision with OMF reconstruction may be more beneficial in recurrent or severe PD which may be incompletely excised by minimally invasive approaches [34, 35]. In addition, excision with OMF reconstruction provides a tension-free off-midline closure and flattens the natal cleft which may mitigate recurrence by decreasing skin maceration, bacterial growth, and hair ingrowth that can occur within a deep natal cleft. By decreasing disease recurrence with a more durable initial operation, we also observed lower rates of reoperation. Similar trends have also been observed in prior studies comparing minimally invasive techniques to excisional approaches [33]. While this adds to the existing data directly comparing minimally invasive treatment approaches with excision and OMF reconstruction in the pediatric population, a longitudinal multi-institutional study with long-term follow-up is needed to validate these findings.

Although patients experienced higher rates of recurrence and reoperation with the EPSiT procedure, they also had lower rates of wound complications compared to the excision/OMF reconstruction cohort. Due to the larger extent of excision and reconstruction with the latter approach, wound complications with excision and OMF reconstruction have been reported in up to 25% of pediatric patients with PD [17, 19, 21]. In the excision/OMF reconstruction cohort, we observed partial wound dehiscence in four patients (22.2%) which did not require reoperation, as well as no cases of wound infection, seroma, or hematoma formation. Two (50%) of these occurred in patients who underwent the Bascom cleft lift flap, and two (50%) of these occurred in patients who underwent the modified Limberg flap. In one patient, early unintentional drain removal led to acute wound dehiscence, while early return to athletic activity in another patient led to delayed wound dehiscence in a previously healed scar. While wound complications are a major limitation to excisional approaches, Limberg or MLF reconstruction have been previously reported as having the lowest relative risk of wound dehiscence when compared to alternative OMF reconstruction techniques [24]. Another major concern surrounding wound complications is the subsequent elevated risk of disease recurrence observed after wound dehiscence [36]. We did observe one case of recurrence after wound dehiscence in the excision/OMF reconstruction cohort, but despite this, the overall recurrence rate in this cohort remained low. Thus, while minimally invasive techniques decreased morbidity due to wound complications in the early post-operative period, it did not significantly alter recurrence rates compared to excision/OMF reconstruction approaches.

We also observed that the EPSiT procedure was able to be performed with a significantly shorter operative time compared to excision with OMF reconstruction. Prior experiences with excision and OMF reconstruction in the pediatric population have reported median operative times ranging from 67 to 92 min [19, 20], while shorter operative times up to 41 min have been reported with minimally invasive procedures [22]. Comparing these two operative techniques, the EPSiT approach was designed to be less extensive and less technically demanding compared to excisional/reconstructive techniques which explains the difference in operative duration [12, 37]. As a result, minimally invasive techniques also subsequently reduce anesthetic exposure, healthcare costs, and resource utilization [38]. However, as the EPSiT procedure requires a pediatric cystoscope and is often performed in the operating room in the pediatric population, additional research is needed to fully evaluate the long-term healthcare costs and utilization associated with disease recurrence and recurrent treatment in patients undergoing minimally invasive treatment.

It is important to note that while these findings demonstrate the benefits and limitations of both the EPSiT procedure and excision/OMF reconstruction, both options remain reasonable treatment options when compared to alternative excisional approaches. Although excision with primary midline closure can excise a larger area of diseased tissue and may shorten healing time compared to open healing by secondary intent, this approach is associated with elevated rates of wound complications (e.g., dehiscence, infection/abscess, seroma, hematoma) up to 65% and recurrence rates ranging from 21 to 37.5% [21, 23, 39, 40]. While excision with open healing by secondary intent or negative wound pressure therapy (NWPT) has been recommended to decrease rates of infectious wound complications, this approach is limited by prolonged healing times ranging from 62 days (NWPT) to three months (open wound packing) [41, 42]. In addition, NWPT requires frequent sponge/dressing changes and may lead to additional complications such as device malfunction or retained wound sponges that can further prolong healing time and increase treatment-related morbidity [42]. Thus, while the limitations of the minimally invasive treatment options and excision/OMF reconstruction are outlined in this study, alternative approaches to wound closure after excision (primary midline closure, NWPT, open wound packing) are not recommended [11, 43].

This comparative study has several limitations. Determination of disease recurrence may be limited by the short-term follow-up duration in this study. Although follow-up duration was similar between the two cohorts allowing for direct comparison of short-term disease recurrence, longer duration of follow-up has been shown to be directly associated with PD recurrence [24, 44, 45]. Thus, the follow-up duration in this study may not fully capture additional recurrences and long-term longitudinal monitoring would be prudent in validating these findings. In addition, although this study was performed at a freestanding children’s hospital which serves as a tertiary referral center in a large urban setting, follow-up data may be limited if patients utilized multiple centers for their care. Thus, this study is also vulnerable to the limitations associated with retrospective single-center studies. The retrospective nature of this study is also subject to selection bias and limits insight into treatment selection. As we observed that the entire patient population and the EPSiT cohort was predominantly female, which may be due to selection bias or a treatment approach preference by sex. As PD predominantly occurs in males, the majority of male patients may have been treated via an alternative approach (e.g., excision with midline primary closure or healing by secondary intent), and were, thus, excluded due to the objective of directly comparing two specific treatment approaches. Thus, a larger prospective randomized trial is needed to mitigate potential confounding which this study type is vulnerable to, as well as to further elucidate the optimal treatment method among patients of varying disease severity.

## Conclusion

The treatment of pilonidal disease in the pediatric population remains challenging as exemplified by high rates of disease recurrence and reoperation. This study compares the two commonly used treatment approaches of excision with off-midline flap reconstruction and minimally invasive sinus tract excision. Compared to the EPSiT procedure, excision with off-midline flap reconstruction was associated with lower rates of recurrence and reoperation, but prolonged operative times and higher rates of wound dehiscence. Prospective randomized longitudinal data are vital in further evaluating the findings observed in this study.

## Data Availability

The datasets generated and/or analyzed are available from the corresponding author on reasonable request.

## Abbreviations

EPSiT: Endoscopic pilonidal sinus treatment
MLF: Modified Limberg flap
NWPT: Negative pressure wound therapy
OMF: Off-midline flap
PD: Pilonidal disease
